# Path Analysis of Factors that Influence Family Caregiver Burden in Dementia Care

**DOI:** 10.1093/geroni/igaf122.3990

**Published:** 2025-12-31

**Authors:** Jiyoung Kim, EunKyo Kim, Minhee Yang, Eunhee Cho, Sinwoo Hwang, Jungwon Cho

**Affiliations:** Yonsei University, Seoul, Republic of Korea; Yonsei University, Seoul, Republic of Korea; Yonsei University, Seoul, Republic of Korea; Yonsei University, Seoul, Republic of Korea; Yonsei University, Seoul, Republic of Korea; Yonsei University, Seoul, Republic of Korea

## Abstract

Dementia, affecting over 55 million people worldwide, demands urgent attention to caregiver burden, but the interplay of contributing factors remains unclear. This study aimed to identify factors from both people living with dementia (PLWD) and their family caregivers that influence caregiver burden, and to examine the interrelationships among these factors. Data were drawn from 178 dyads of community-dwelling PLWD and their family caregivers in Korea. Family caregiver burden was assessed through caregivers’ subjective health and perceived stress related to their family member’s behavioral and psychological symptoms of dementia (BPSD). The analysis was grounded in the Stress Process Model, which provided a theoretical framework for examining caregiver burden. We modified the model by repositioning BPSD as the primary stressors of caregiver burden and additionally incorporating PLWD factors as antecedents to BPSD. Path analysis was conducted using JASP 0.19.3.0, and model fit was evaluated using multiple fit indices. Agitation and depression—key BPSD of PLWD—were negatively associated with both caregivers’ subjective health and stress levels. These associations were also indirectly influenced by PLWD’s age. Furthermore, caregivers’ sex, age, and economic status were directly associated with their subjective health. Neither caregiving hours nor caregiving duration significantly influenced caregiver burden. These findings underscore the significant impact of agitation and depression on caregiver burden and indicate that when BPSD manifest, the burden experienced by caregivers should also be addressed. Thus, tailored dementia interventions addressing these factors need to be developed to mitigate caregiver burden and improve outcomes for both PLWD and their families.

